# Mesenchymal stem cells-derived therapies for subarachnoid hemorrhage in preclinical rodent models: a meta-analysis

**DOI:** 10.1186/s13287-022-02725-2

**Published:** 2022-01-29

**Authors:** Jialin He, Jianyang Liu, Yan Huang, Ziwei Lan, Xiangqi Tang, Zhiping Hu

**Affiliations:** 1 Department of Neurology, The Second Xiangya Hospital of Central South University, Changsha, 410011 Hunan People’s Republic of China; 2 National Health Commission Key Laboratory of Birth Defect for Research and Prevention, Hunan Provincial Maternal and Child Health Care Hospital, Changsha, 410008 Hunan People’s Republic of China

**Keywords:** Subarachnoid hemorrhage, Mesenchymal stem cell, Extracellular vesicles, Meta-analysis, Animal model

## Abstract

**Background:**

Mesenchymal stem cells (MSCs) and MSCs-derived extracellular vesicles (EVs) have emerged as potential novel therapies for subarachnoid hemorrhage (SAH). However, their effects remain incompletely understood. We aim to comprehensively evaluate the effect of MSCs-derived therapies in rodent models of SAH.

**Methods:**

We searched PubMed, EMBASE, and Web of Science up to September 2021 to identify studies that reported the effects of MSCs or MSCs-derived EVs in a rodent SAH model. Neurobehavioral score was extracted as the functional outcome, and brain water content was measured as the histopathological outcome. A random-effects model was used to calculate the standardized mean difference (SMD) and confidence interval (CI).

**Results:**

Nine studies published from 2018 to 2021 met the inclusion criteria. Studies quality scores ranged from 5 to 10, with a mean value of 7.22. Our results revealed an overall positive effect of MSCs and MSCs-derived EVs on the neurobehavioral score with a SMD of − 2.21 (95% CI − 3.14, − 1.08; *p* < 0.0001). Meanwhile, we also found that MSCs and MSCs-derived EVs reduced brain water content by a SMD of − 2.09 (95% CI − 2.99, − 1.19; *p* < 0.00001). Significant heterogeneity among studies was observed, further stratified and sensitivity analyses did not identify the source of heterogeneity.

**Conclusions:**

Our results suggested that MSCs-derived therapies prominently improved functional recovery and reduced brain edema in the rodent models of SAH. Notably, the limitations of small sample size should be considered when interpreting the results, and large animal studies and human trials are needed for further investigation.

**Supplementary Information:**

The online version contains supplementary material available at 10.1186/s13287-022-02725-2.

## Introduction

Spontaneous subarachnoid hemorrhage (SAH) is mainly due to aneurysm rupture and accounts for 9.7% of all strokes in 2019, affecting eight in 100,000 individuals each year [1, 2]. Despite SAH being less common than ischemic stroke and intracerebral hemorrhage, its young mean age incidence and high mortality also contribute to a remarkable health burden on society comparable with that of ischemic stroke and intracerebral hemorrhage [3].

Current treatment strategies for SAH include rebleeding prevention via surgery and cerebral vasospasm treatment [4]. However, the optimal clinical therapeutic regimen of SAH remains a challenge for clinicians due to its complicate brain injury mechanism. On the one hand, within the first 72 h, the increased intracranial pressure contributes to early brain injury (EBI), including global cerebral ischemia and edema, blood–brain barrier (BBB) breakdown, and subarachnoid blood toxicity [5]. On the other hand, delayed cerebral vasospasm (CVS) and delayed cerebral ischemia (DCI) are the two main devastating complications at a delayed phase [6, 7]. Notably, the high-grade cerebral edema in early stage is identified to be an independent predictor of DCI and unfavorable clinical outcome [8]. Mechanically, inflammatory activity, oxidative stress, and apoptosis are throughout the entire course of SAH [9, 10].

In recent years, researchers raised the concern about the beneficial effect of stem cell therapy in SAH [11]. The easy accessibility and potent paracrine activities have made mesenchymal stem cells (MSCs) an increasingly popular candidate for the treatment of SAH in comparison with other stem cell types. In most studies, MSCs and MSCs-derived extracellular vesicles (EVs) exhibited the ability to inhibit neuroinflammation, reduce BBB destruction, and ameliorate neurological deficits in the animal models of SAH [12, 13]. By contrast, it was also reported that MSC-derived EVs were unable to improve the neurologic deficit in animals subjected to SAH [14].

Although Ghonim et al. have concluded the potential advantages of MSCs therapy in animals suffering from induced SAH through literature review in 2016 [15], there is no meta-analysis to evaluate the quality of preclinical studies and synthesize evidence on the effects of MSC-derived therapies in SAH. The aim of our study is to assess the efficacy of MSC-derived therapies on the behavioral and pathological outcomes of experimental SAH rodents, to provide support for the future clinical trial design of the MSCs treatment following SAH.

## Methods

### Data sources and search strategy

The following online databases were searched for experimentally controlled studies of the effect of MSCs-derived therapies on SAH models: PubMed, EMBASE, and Web of Science (all until September 30, 2021). The following search terms were used: ((((Subarachnoid hemorrhage) OR (Subarachnoid Hemorrhage, Aneurysmal)) OR (Aneurysmal Subarachnoid Hemorrhage)) OR (SAH)) AND (((((mesenchymal stem cell) OR (mesenchymal stromal cell)) OR (MSC)) OR (exosome)) OR (extracellular vesicle)). The reference lists of the included studies were also searched to identify other relevant articles.

### Inclusion and exclusion criteria

Studies were included if they met the following criteria: (1) SAH models was induced in rodent animals; (2) the effect of unmodified MSCs or MSCs-derived EVs was tested in at least one experimental group; (3) studies provided adequate data on neurobehavioral score or brain water content; (4) experimental studies were presented in original research and published in peer-reviewed journals; and (5) studies were published in English.

The exclusion criteria were as follows: (1) studies that did not include in vivo testing; (2) the outcome did not include the neurobehavioral score or brain water content; (3) studies that published as clinical research, review, and conference abstract.

### Study selection

Duplicate articles were automatically excluded from EndNote and the remaining studies were selected manually. The title and abstract of the relevant articles were reviewed to identify eligible papers. Full-text articles were then obtained and reviewed thoroughly for the final eligibility according to the inclusion and exclusion criteria stated above. Two investigators conducted the study selection independently. Disagreements were addressed by discussion with a third reviewer.

### Data extraction

Two independent authors extracted data from studies based on author; year; animal species and sex; anesthetic types; method of SAH induction; intervention (MSCs source, type of MSCs-derived therapies, and the route and time of administration); sample size; assessment time; functional outcome (neurobehavioral score); and histopathological outcome (brain water content).

The mean and standard deviation (SD) of neurobehavioral score and brain water content in the treatment group and control group were extracted independently by two investigators. If SD was not reported, it was calculated through multiplying the standard error (SE) by the square root of the sample size. For studies that had not shown the corresponding results, the GetData Graph Digitizer software (version 2.26; GetData; http://getdata-graph-digitizer.com/download.php/) was used to extract data from the graphics. When the outcomes were measured at different time points, only the data from the final point was extracted. Disagreement between two investigators was solved by checking the data in the publications together. Moreover, if the data of multiple brain slices were reported in histopathological outcomes, only the data of ipsilateral basal ganglia were extracted.

### Quality assessment

The methodological quality of each study was evaluated by two independent researchers according to Collaborative Approach to Meta-Analysis and Review of Animal Data from Experimental Studies (CAMARADES) 10-item checklist with minor modifications [16]. One point was given for each of the following criteria: (1) peer-reviewed publication; (2) sample size calculation; (3) randomized treatment allocation; (4) blinded induction of SAH; (5) blinded assessment of outcome; (6) suitable animal models; (7) use of anesthetic without marked intrinsic neuroprotective activity; (8) compliance with animal welfare regulations; (9) statements describing temperature control; and (10) declarations of potential conflicts of interest. We defined studies that scored < 5 points as low quality, and those that scored ≥ 5 points as high quality.

### Statistical analysis

Stata 15.1 (StataCorp, College Station, TX, USA) and Cochrane Review Manager 5.3 (Cochrane Collaboration; www.cochrane.org/) were used to perform data analyses. The combined effect size was calculated as standardized mean difference (SMD) with 95% confidence interval (CI) between treatment group and control group. Forest plots were generated to display the SMD and 95% CI of each study, and the pooled mean difference by combining all studies. All statistical tests were two-sided, and a *p*-value of less than 0.05 was considered statistically significant.

The *I*^2^ statistic was applied to estimate the total variation attributed to heterogeneity among studies. The values of *I*^2^ that ranged from 0 to 40%, 30–60%, 50–90%, and 75–100% were defined as “low,” “moderate,” “substantial,” and “considerable” heterogeneity, respectively [17]. The Hedge’s random-effects model was adopted to comprehensively estimate the effect size because of the substantial heterogeneity. Sensitivity and stratified analyses were performed to identify the source of the heterogeneity and to investigate the other potential confounding factors. A funnel plot was applied to check for publication bias [18], the asymmetry of which was evaluated using Egger’s test and the trim-and-fill method [19].

## Results

### Study selection process

**Fig. 1:**
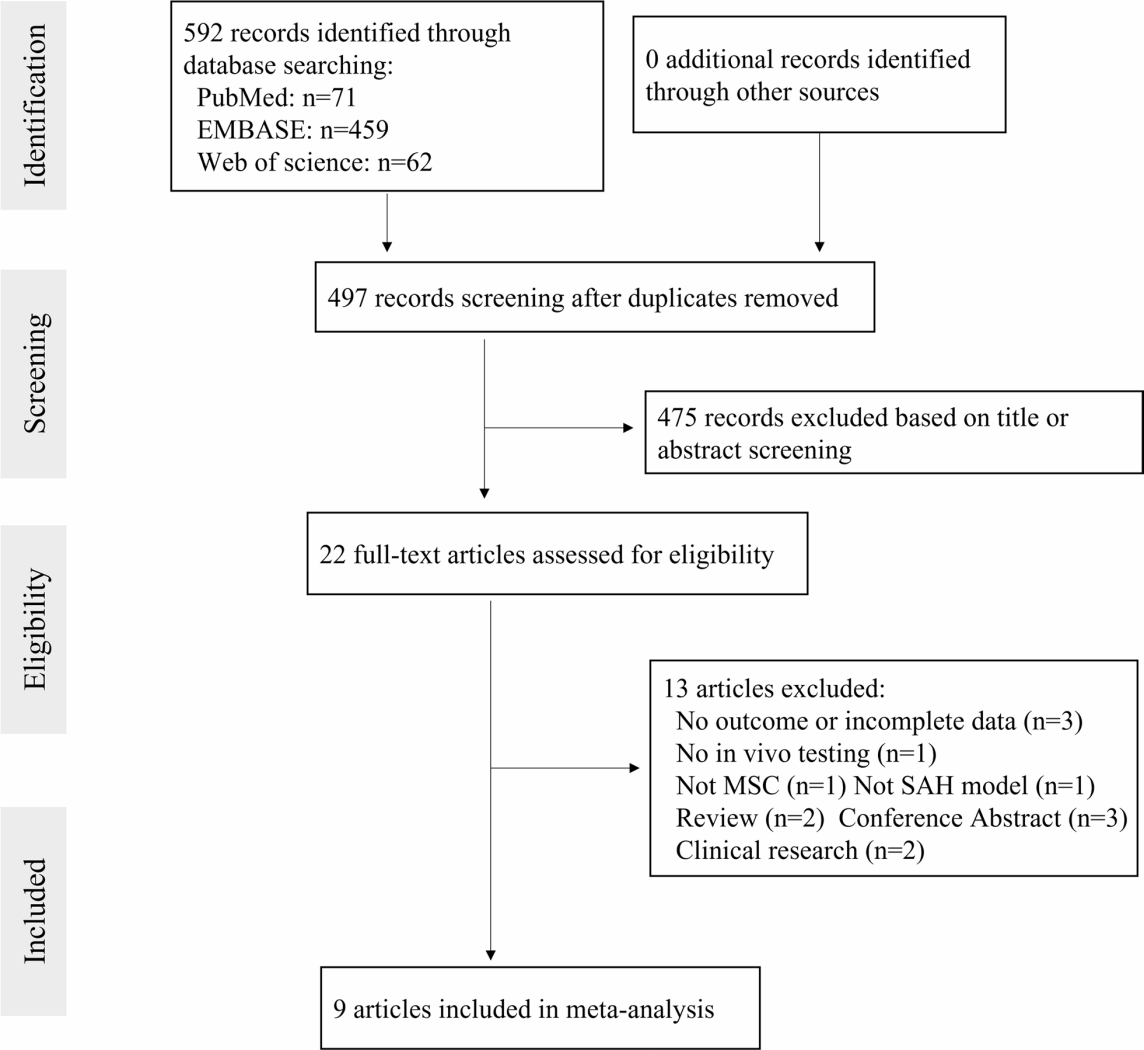
PRISMA flow diagram for review and selection process of studies included in meta-analysis of mesenchymal stem cells-derived therapies in rodent models of subarachnoid hemorrhage

Our study was conducted and reported in compliance with Preferred Reporting Items for Systematic Review and Meta-Analyses (PRISMA) guidelines [20]. The process of study selection is shown in Fig. 1. The literature search identified 592 potential studies at the primary retrieval: 71 records in PubMed, 459 in EMBASE, and 62 in Web of science. After review and exclusion, twenty-two full-text articles remained, which were then assessed for the inclusion eligibility. And thirteen of them were excluded because of the following reasons: no outcome or incomplete data (*n* = 3), no in vivo testing (*n* = 1), not MSCs (*n* = 1), not SAH model (*n* = 1), review articles (*n* = 2), conference abstract (*n* = 3), and clinical research articles (*n* = 2). Finally, our study included nine articles published from 2018 to 2021 that met the inclusion criteria [12–14, 21–26].

### Study characteristics

The main characteristics of the included studies are given in Table 1. All animal models were established in mice and rats. Male animals were used in most of the studies, while one study did not mention the gender of animals. Regarding the anesthetic drugs, three studies used pentobarbital, two studies used chloral hydrate, three studies was done with isoflurane, and one study did not report the types of anesthetic drugs. Five studies used endovascular perforation to induce the SAH model, the remaining four studies induced the model with autogenous blood injection. The tissue sources of MSCs included bone marrow and umbilical cord obtained from human or rodents. MSCs-derived EVs were adopted in six studies, while MSCs were used in the remaining three studies. Those MSCs and MSCs-derived EVs were transplanted via intravenous (IV) injection in most studies. Most studies performed the MSCs or MSCs-derived EVs treatment after the induction of SAH model, only one study was done before the SAH induction and included repeated injections. Additionally, the outcome assessment time points of the studies varied significantly; most of them were completed within 72 h after SAH induction, while two studies lasted up to 21 days. Functional outcome was assessed in nine studies using Garcia score, behavior score, and adhesive removal task. Seven studies evaluated the histopathological outcome by detecting the brain water content.

**Table 1:** Characteristics of the included studies

References	Animal, sex	Anesthetic	Method of SAH	MSCs source	Type of MSCs derived therapy	Time of administration	Tre(*n*)/con(*n*)	Assessment time	Outcome measure
Chen [21]	SD rat, male	Pentobarbital, IP	Autogenous blood	Human UC-MSCs	UC-MSCs, 2 × 10^5^, ICV	− 1, 10 days post SAH	16/16	21 days	Garcia score and beam balance test (higher is better)
Gao [22]	SD rat, male	Chloral hydrate, IP	Endovascular perforation	BM-MSCs	BM-MSCs-derived EVs, 100 ug, IV	1 h post SAH	12/12; 6/6	48 h	Garcia score (higher is better); BWC (lower is better)
Han [12]	SD rat, male	Isoflurane, inhalation	Endovascular perforation	Rat BM-MSCs	BM-MSCs-derived EVs, 100 ug/500 ul, IV	10 min post SAH	5/5; 5/5	48 h	Garcia score (higher is better); BWC (lower is better)
Lai [14]	C57BL/6 mice, male	Chloral hydrate, IP	Autogenous blood	Mice BM-MSCs	BM-MSCs-derived EVs, IV	22 h post SAH	18/18; 6/6	24 h	Garcia score (higher is better); BWC (lower is better)
Liu [23]	SD rat, male	Pentobarbital, IP	Endovascular perforation	Rat BM-MSCs	BM-MSCs, 3 × 10^6^, IV	1 h post SAH	12/12; 6/6	72 h	Garcia score (higher is better); BWC (lower is better)
Liu [24]	SD rat, NR	NR	Autogenous blood	Human UC-MSCs	UC-MSCs-derived EVs, 100 ug/ml, IV	NR	6/6; 6/6	24 h	Behavior score (lower is better); BWC (lower is better)
Nijboer [13]	Wistar rat, male	Isoflurane, inhalation	Endovascular perforation	SD rat BM-MSCs	BM-MSCs, 1.5 × 10^6^, IN	6 days post SAH	13/10	21 days	Adhesive removal task (lower is better)
Xiong [25]	SD rat, male	Isoflurane, inhalation	Endovascular perforation	Rat BM-MSCs	BM-MSCs-derived EVs, 200 ug/200 ul, IV	1 h post SAH	5/5; 5/5	24 h	Garcia score (higher is better); BWC (lower is better)
Zhao [26]	SD rat, male	Pentobarbital, IP	Autogenous blood	Human UC-MSC	UC-MSCs-derived EVs, 400 ug/200 ul, IV	1 h post SAH	5/5; 5/5	24 h	Garcia score (higher is better); BWC (lower is better)

*SAH* subarachnoid hemorrhage, *MSCs* mesenchymal stem cells, *Tre* treatment, *Con* control, *IP* intraperitoneal, *NR* not recorded, *UC-MSCs* umbilical cord mesenchymal stem cells, *BM-MSCs* bone marrow mesenchymal stem cells, *EVs* extracellular vesicles, *ICV* intracerebroventricular, *IV* intravenous, *IN* intranasal, *BWC* brain water content

### Study quality

The quality score of the included studies varied from 5 to 10 (mean 7.22), all of them were regarded as high methodological quality (≥ 5) studies. All studies were published in peer-reviewed journals, used suitable animal models, stated compliance with animal welfare regulations, and declared potential conflicts of interest. Only one (11.11%) study reported a sample size calculation, eight (88.89%) studies allocated animals to treatment or control randomly, and two (22.22%) studies reported a blinded induction of SAH. The numbers of studies that used a blinded assessment to evaluate outcomes, used anesthetics without marked intrinsic neuroprotective properties, and reported describing control of temperature were seven (77.78%), eight (88.89%), and three (33.33%), respectively. The details of quality index are given in Table 2.

**Table 2:** Methodological quality of nine studies included in the meta-analysis

Study	(1)	(2)	(3)	(4)	(5)	(6)	(7)	(8)	(9)	(10)	Total
Chen [21]	√		√		√	√	√	√	√	√	8
Gao [22]	√		√	√	√	√	√	√		√	8
Han [12]	√		√		√	√	√	√		√	7
Lai [14]	√				√	√	√	√		√	6
Liu [23]	√		√		√	√	√	√		√	7
Liu [24]	√		√			√		√		√	5
Nijboer [13]	√	√	√	√	√	√	√	√	√	√	10
Xiong [25]	√		√		√	√	√	√	√	√	8
Zhao [26]	√		√			√	√	√		√	6

(1) peer-reviewed publication; (2) sample size calculation; (3) randomized treatment allocation; (4) blinded induction of SAH; (5) blinded assessment of outcome; (6) suitable animal models; (7) use of anesthetic without marked intrinsic neuroprotective activity; (8) compliance with animal welfare regulations; (9) statements describing temperature control; (10) declarations of potential conflicts of interest

### Global estimates of efficacy

**Fig. 2:**
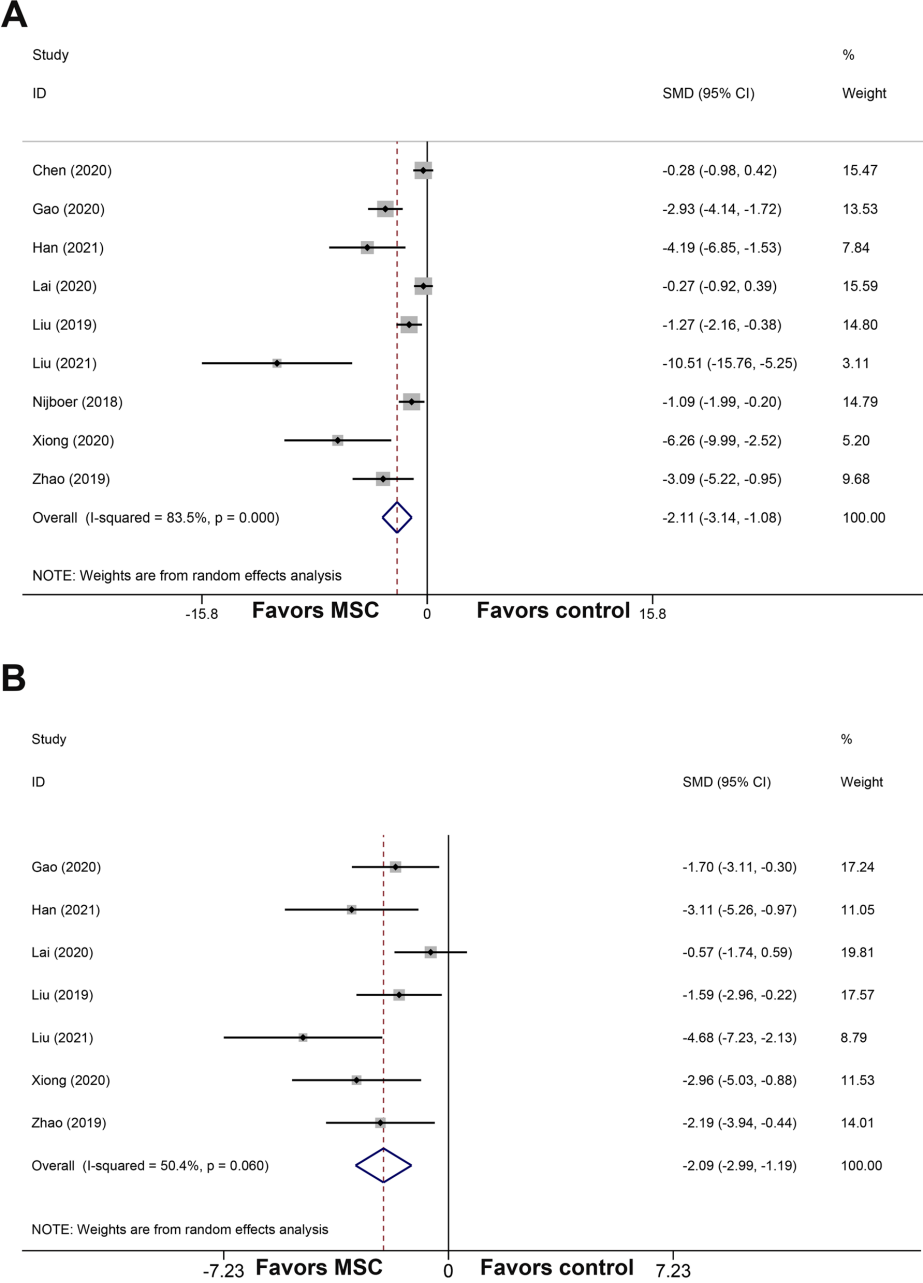
Forest plot shows the mean effect size and 95% confidence interval (CI) for neurobehavioral score (**A**) and brain water content (**B**) between MSCs-derived therapies treatment group and control group in all studies. *MSCs* mesenchymal stem cells; *SMD* standardized mean difference

All studies reported the neurobehavioral score. The pooled analysis showed that MSCs and MSCs-derived EVs could improve neurobehavioral outcome apparently when compared with the control group (SMD =  − 2.11; 95% CI − 3.14, − 1.08; Fig. 2A). The heterogeneity of neurobehavioral outcome among comparisons was statistically significant (*I*^2^ = 83.5%, *p* = 0.000).

In addition, seven studies analyzed the brain water content. MSCs and MSCs-derived EVs showed a significant reduction in the brain water content by an SMD of − 2.09 (95% CI − 2.99, − 1.19; Fig. 2B), with a statistically significant heterogeneity (*I*^2^ = 50.4%, *p* = 0.06).

### Sensitivity analysis

**Fig. 3:**
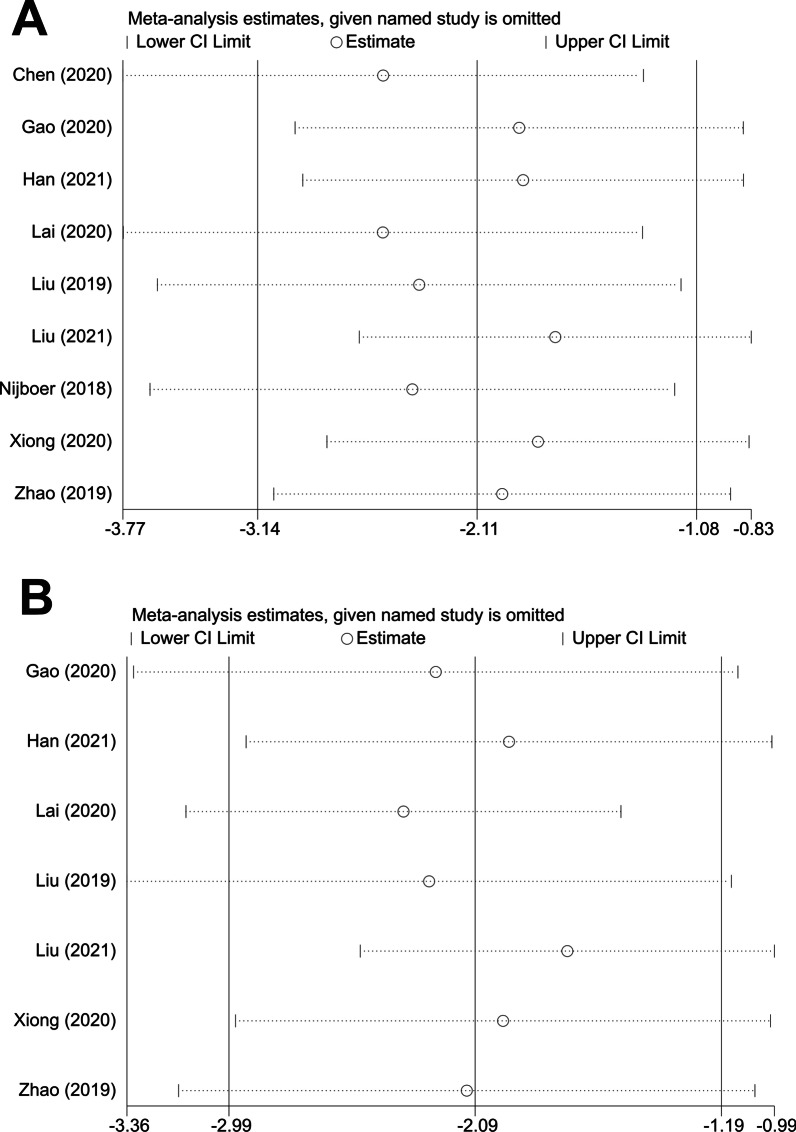
Sensitivity analysis of the studies included in neurobehavioral score (**A**) and brain water content (**B**)

To evaluate the stability of the results, we further performed a sensitivity analysis through the sequential omission of each study. For the pooled SMD, neither neurobehavioral outcome nor histopathological outcome were significantly affected by any study (Fig. 3A, B).

### Stratified analysis

Details of the stratified analysis on neurobehavioral score and brain water content are given in Tables 3 and 4, respectively.

**Table 3:** Stratified meta-analysis of heterogeneity on neurobehavioral score

Categories	No. of studies	Pooled SMD (95% CI)	*p* value	Heterogeneity test			Between groups *p* value
				*Q* statistics	*I*^2^	*p* value	
*Anesthetic type*							0.006
Pentobarbital	3	− 1.20 (− 2.41, 0.00)	0.05	7.59	74%	0.02	
Chloral hydrate	2	− 1.55 (− 4.15, 1.06)	0.24	14.36	93%	0.0002	
Isoflurane	3	− 3.47 (− 6.62, − 0.32)	0.03	10.83	82%	0.004	
NR	1	− 10.51 (− 15.76, − 5.25)	< 0.0001	NA	NA	NA	
*Method of SAH*							0.47
Autogenous blood	4	− 1.67 (− 3.27, − 0.07)	0.04	20.44	85%	0.0001	
Endovascular perforation	5	− 2.42 (− 3.67, − 1.16)	0.0002	15.67	74%	0.003	
*MSCs species*							0.25
Non-xenogeneic	5	− 1.64 (− 2.79, − 0.49)	0.005	18.14	78%	0.001	
Xenogeneic	3	− 3.71 (− 7.64, 0.22)	0.06	19.58	90%	< 0.0001	
NR	1	− 2.93 (− 4.14, − 1.72)	< 0.00001	NA	NA	NA	
*MSCs type*							0.40
UC-MSC	3	− 3.71 (− 7.64, 0.22)	0.06	19.58	90%	< 0.0001	
BM-MSC	6	− 1.96 (− 3.10, − 0.82)	0.0008	27.50	82%	< 0.0001	
*Therapy type*							0.01
MSCs	3	− 0.82 (− 1.46, − 0.19)	0.01	3.58	44%	0.17	
MSCs-derived EVs	6	− 3.76 (− 5,92, − 1.60)	0.0006	42.18	88%	< 0.00001	
*Delivery route*							0.005
IV	7	− 3.03 (− 4.57, − 1.50)	0.0001	42.22	86%	< 0.00001	
ICV	1	− 0.28 (− 0.98, 0.42)	0.43	NA	NA	NA	
IN	1	− 1.09 (− 1.99, − 0.20)	0.02	NA	NA	NA	
*Administration time*							< 0.0001
Pre-SAH	1	− 0.28 (− 0.98, 0.42)	0.43	NA	NA	NA	
Post-SAH	7	− 2.10 (− 3.18, − 1.02)	0.0001	30.53	80%	< 0.0001	
NR	1	− 10.51 (− 15.76, − 5.25)	< 0.0001	NA	NA	NA	
*Assessment time*							0.007
≤ 72 h	7	− 3.03 (− 4.57, − 1.50)	0.0001	42.22	86%	< 0.00001	
> 72 h	2	− 0.64 (− 1.42, 0.15)	0.11	1.96	49%	0.16	

*SMD* standardized mean difference, *CI* confidence interval, *SAH* subarachnoid hemorrhage, *MSCs* mesenchymal stem cells, *NR* not recorded, *UC-MSCs* umbilical cord mesenchymal stem cells, *BM-MSCs* bone marrow mesenchymal stem cells, *EVs* extracellular vesicles, *ICV* intracerebroventricular, *IV* intravenous, *IN* intranasal, *NA* not available

**Table 4:** Stratified meta-analysis of heterogeneity on brain water content

Categories	No. of studies	Pooled SMD (95% CI)	*p* value	Heterogeneity test			Between groups *p* value
				*Q* statistics	*I*^2^	*p* value	
*Anesthetic type*							0.03
Pentobarbital	2	− 1.82 (− 2.90, − 0.74)	0.001	0.28	0%	0.60	
Chloral hydrate	2	− 1.07 (− 2.17, 0.03)	0.06	1.47	32%	0.22	
Isoflurane	2	− 3.03 (− 4.52, − 1.54)	< 0.0001	0.01	0%	0.92	
NR	1	− 4.68 (− 7.23, − 2.13)	0.0003	NA	NA	NA	
*Method of SAH*							0.88
Autogenous blood	3	− 2.24 (− 4.39, − 0.09)	0.04	9	78%	0.01	
Endovascular perforation	4	− 2.06 (− 2.88, − 1.24)	< 0.00001	2.35	0%	0.50	
*MSCs species*							0.52
Non-xenogeneic	4	− 1.81 (− 3.00, − 0.62)	0.003	6.50	54%	0.09	
Xenogeneic	2	− 3.25 (− 5.67, − 0.84)	0.008	2.49	60%	0.11	
NR	1	− 1.70 (− 3.11, − 0.30)	0.02	NA	NA	NA	
*MSCs type*							0.24
UC-MSCs	2	− 3.25 (− 5.67, − 0.84)	0.008	2.49	60%	0.11	
BM-MSCs	5	− 1.72 (− 2.60, − 0.83)	0.0002	6.55	39%	0.16	
*Therapy type*							0.46
MSCs	1	− 1.59 (− 2.96, − 0.22)	0.02	NA	NA	NA	
MSCs-derived EV or exosome	6	− 2.26 (− 3.36, − 1.15)	< 0.0001	11.95	58%	0.04	

*SMD* standardized mean difference, *CI* confidence interval, *SAH* subarachnoid hemorrhage, *MSCs* mesenchymal stem cells, *NR* not recorded, *UC-MSCs* umbilical cord mesenchymal stem cells, *BM-MSCs* bone marrow mesenchymal stem cells, *EVs* extracellular vesicles, *NA* not available

For the neurobehavioral score, we stratified the data by anesthetic drugs, the studies that used isoflurane showed a higher effect size than others (*p* = 0.006, Additional file 1: Fig. S1), and the results of studies that used pentobarbital and chloral hydrate were not statistically significant. The methods used to induce SAH model exhibited no significant differences in the estimation of effect size (*p* = 0.47, Additional file 2: Fig. S2). Neither the species of MSCs nor the types of MSCs had distinction in the estimation of effect size (*p* = 0.25, Additional file 3: Fig. S3; *p* = 0.40, Additional file 4: Fig. S4). Interestingly, MSCs-derived EVs seemed to be more effective in the improvement of neurobehavioral outcome than MSCs (*p* = 0.01, Additional file 5: Fig. S5). Concerning the delivery route, IV injection displayed a higher effect size than intranasal (IN) injection, and the result of intracerebroventricular (ICV) injection was not statistically significant (*p* = 0.005, Additional file 6: Fig. S6). And MSCs or MSCs-derived EVs that administrated after SAH induction was more favorable than those administrated before the SAH induction (*p* < 0.0001, Additional file 7: Fig. S7). Finally, the studies that assessed neurobehavioral score within 72 h after SAH induction exhibited a higher effect size than those assessed more than 72 h (*p* = 0.007, Additional file 8: Fig. S8).

Meanwhile, for the brain water content, our stratified analysis found that the studies used isoflurane displayed the greatest efficacy, followed by those used pentobarbital and then chloral hydrate (*p* = 0.03, Additional file 9: Fig. S9). The method of SAH model induction showed no significant differences in the estimation of effect size (*p* = 0.88, Additional file 10: Fig. S10). And the species and types of MSCs had no impact on the estimation of effect size (*p* = 0.52, Additional file 11: Fig. S11; *p* = 0.24, Additional file 12: Fig. S12). Additionally, there was no significant difference between the MSCs group and MSCs-derived EVs group (*p* = 0.46, Additional file 13: Fig. S13).

### Publication bias

**Fig. 4:**
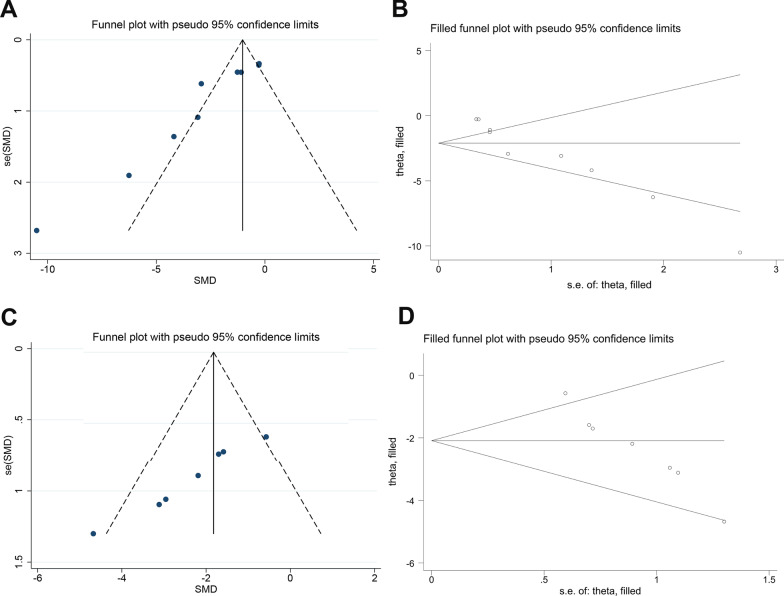
Evaluation of publication bias. Funnel plots for neurobehavioral score (**A**) and brain water content (**C**). Each funnel plot displays all studies in one plot with SMD as the *x*-value and 1/*n* as the *y*-value. **B** and **D** Trim-and-fill method was used to evaluate the missing studies in neurobehavioral score and brain water content. *SMD* standardized mean difference

Visual inspection of the funnel plot suggested conspicuous publication bias for the neurobehavioral score (Fig. 4A), and the results of the Egger test suggested the same comments (*p* < 0.001). We then used the trim-and-fill method to recalculate the pooled estimation with addition of missing studies. However, the overall results were not significantly changed (Fig. 4B), indicating no “missing” studies.

For the brain water content, although the funnel plot was approximately symmetrical (Fig. 4C), the Egger test indicated significant publication bias (*p* < 0.001). After adopting the trim-and-fill correction for the brain water content, the estimated value remained unchanged (Fig. 4D).

## Discussion

### Summary of evidence

Our meta-analysis of nine studies provided a comprehensive summary regarding the effect of MSCs-derived therapies on the rodent model of SAH. Pooled analyses confirmed that MSCs-derived therapies could improve neurological deficit and reduce brain edema in the preclinical rodent models of SAH. Therefore, the present meta-analysis provides significant clues for human clinical trials on MSCs-derived therapies. However, due to the limited number of studies, more studies are needed to prove the neuroprotective effect of MSCs-derived therapies in experimental SAH.

### Possible mechanisms of the MSCs-derived therapies in SAH

Although the neuroprotective role of MSCs-derived therapies in ischemic stroke and intracerebral hemorrhage has been well-accepted, their therapeutic potential is only gradually explored in SAH. It is well-recognized that MSCs and MSCs-derived EVs exhibited anti-inflammation and anti-apoptosis properties in SAH animal models [11]. MSCs administration can reduce inflammatory cytokines production, promote microglia polarization, inhibit neural cell apoptosis, ameliorate cerebral edema, and promote functional recovery significantly [12, 21, 27, 28]. Other data suggested that the administration of MSCs improved the structural integrity of cerebral tissues and arterial wall in SAH induced rats [29]. There was also reported that BM-MSCs regulated the activation of astrocytes and protected BBB in SAH models [30].

Recently, EVs-mediated miRNA transfer has been proved to play an important role in the SAH models by a number of studies. Among these studies, EVs-mediated transfer of miRNA-21-5p, miRNA-26b-5p, and miRNA-129-5p from MSCs to neurons were able to alleviate neuroinflammation and reduce apoptosis in the SAH animal models [22, 24, 25]. Another study found that human umbilical cord mesenchymal stem cells (UC-MSCs)-derived miRNA-206-knockdown EVs had a better neuroprotective effect on the SAH-induced EBI compared to the treatment with simple EVs, as the miRNA-206-knockdown EVs could upregulate the expression of brain derived neurotrophic factor (BDNF) [26].

### Interpretation of the stratified analysis

Our stratified analysis revealed that animals anesthetized with isoflurane responded better to the MSCs-derived therapies than those anesthetized with chloral hydrate and pentobarbital in terms of the neurobehavioral score and brain water content. Although the neuroprotective effects of isoflurane, phenobarbital, and chloral hydrate in animal models of ischemic stroke and intracerebral hemorrhage have been well-reported [31–34], their role in the SAH animal models was ambiguous. One such study found that isoflurane significantly suppressed post-SAH apoptosis and cerebral inflammation [35], while another study demonstrated that the use of isoflurane and chloral hydrate resulted in deterioration of respiratory parameters and increased brain water content [36]. Therefore, the potential effect of anesthetic should not be ignored when the results are interpreted.

The endovascular perforation model and autologous blood injection model are the most commonly used animal models of SAH [37, 38]. The endovascular puncture model is mainly performed in rats and mice, and rabbits, dogs, rats, and mice were frequently used animals in the autologous blood injection model. In general, the mortality rate of the autologous blood injection model is lower than that of the endovascular perforation model. The amount of the blood injected into the subarachnoid space is always fixed in the autologous blood injection model, while the extent of the hemorrhage is variable after puncture in the endovascular perforation model [39]. Our results observed no difference in the effect size of SAH models in both of the neurobehavioral score and brain water content, indicating that these two models are suitable for the preclinical studies of MSCs transplantation.

The MSCs used in the included studies were bone marrow mesenchymal stem cells (BM-MSCs) and UC-MSCs obtained from human or rodents. According to our stratified analysis, neither of the species and the types of the MSCs had impact on the effect size in terms of the neurobehavioral score and brain water content, suggesting that the beneficial effects of MSCs-derived therapies in SAH animal models were probably not dependent on the source of MSCs.

It is well accepted that MSCs exert advantageous effects mainly through their potent paracrine activities [40]. MSCs-derived EVs possess the ability to cross the BBB and the capacity of targeted delivering gene drugs, which seem to make the therapeutic potential of MSCs-derived EVs more pronounced relative to MSCs in the central nervous system disease [41]. Interestingly, we found that the therapy type contributed to apparent differences in the neurobehavioral score, but not for brain water content. MSCs-derived EVs appeared to show more efficacy than MSCs in the promotion of neurological function recovery. However, the numbers of included studies were too small, larger well-designed preclinical studies are needed to explore these issues in-depth.

The route and timing of administration are another two factors that influence the cell therapy efficiency [11]. There was no experimental study compared the distinction in treatment efficacy among different transplantation routes and time previously. Our study found that IV injection was more effective than IN injection in the amelioration of neurobehavioral outcome. Moreover, MSCs and MSCs-derived EVs administrated after the SAH induction exhibited better neurobehavioral outcome than those injected before SAH induction. However, small sample sizes diminished the robustness of the data.

As stated before, the initial hemorrhage severity in the early stage and secondary brain injury of the late stage are the major determinants of outcomes after SAH [42], but evidence regarding the difference in the efficacy of MSCs for EBI and late brain injury after SAH remains lacking. Therefore, we performed stratified analysis about the time of assessment, and found a negative correlation between the effect size and assessment time in the neurobehavioral score. These findings implied that the EBI caused by SAH induction may respond better to MSCs-derived therapies in comparison with that in the delayed stage. But the number of studies included for late endpoints was small, this comparison needs to be verified by more investigations.

Overall, significant differences between groups were found in some stratified analyses, but the source of heterogeneity was not identified according to the stratified analysis. It should be also noticed that the subgroup analysis only generates hypothesis rather than confirming them.

### Clinical perspective

To date, no clinical trial has been carried out on the treatment of SAH with MSCs-derived therapies, and only one case report using allogeneic BM-MSCs transplantation on human for treatment of high-grade aneurysmal SAH was documented [43]. There is significant work to be done for the future clinical translation. First, most research subjects are rats and mice, and they cannot simulate well the physiological conditions of humans suffered from SAH. Therefore, primates’ models should be established to obtain more results. Second, the dose of MSCs or MSCs-derived EVs is typically the topic of concern when they are applied in clinical situations. The isolation method of EVs from MSCs varied in different studies, making the dose comparison between MSCs and EVs hard. In order to identify the best dose of MSCs and MSCs-derived EVs, the standardization of the isolation method of EVs from MSCs may be necessary. Third, MSCs and MSCs-derived EVs were administrated within 1 h after SAH induction in most preclinical studies, but in fact, most SAH patients would be treated more than 1 h after onset. To determine the optimal transplantation time of MSCs products in SAH patients, more animal studies in line with the clinical settings and more clinical trials need to be carried out. Accordingly, as the observed beneficial effects of MSCs and MSCs-derived EVs in the SAH animal models, the clinical translation of MSCs-derived therapies for the treatment of SAH is promising.

### Limitations

Several potential limitations to our meta-analysis should be considered. First, although we performed stratified and sensitivity analyses, the heterogeneity among studies could not be remarkably reduced. This may influence the stability of the results. Second, our research only included the available data, some negative results were less likely to have been published, which could have introduced publication bias. Third, our meta-analysis was limited by a small data set (only nine publications), further studies with large sample sizes are warranted to provide sufficient evidence about the effect of MSCs-derived therapies on SAH and to guide their application in clinical settings.

## Conclusion

Based on the data of our meta-analysis, MSCs-derived therapies showed neuroprotection compared with control group, by evaluating the treatment outcomes including neurobehavioral score and brain water content. However, more large animal studies and human trials are needed for further investigation.

## Supplementary Information

**Additional file 1: Fig. S1.** Subgroup analysis by anesthetic drugs for the neurobehavioral score.

**Additional file 2: Fig. S2.** Subgroup analysis by the method of SAH induction for the neurobehavioral score.

**Additional file 3: Fig. S3.** Subgroup analysis by the source of MSCs for the neurobehavioral score.

**Additional file 4: Fig. S4.** Subgroup analysis by the type of MSCs for the neurobehavioral score.

**Additional file 5: Fig. S5.** Subgroup analysis by the type of MSCs-derived therapies for the neurobehavioral score.

**Additional file 6: Fig. S6.** Subgroup analysis by delivery route for neurobehavioral score.

**Additional file 7: Fig. S7.** Subgroup analysis by administration time for the neurobehavioral score.

**Additional file 8: Fig. S8.** Subgroup analysis by assessment time for the neurobehavioral score.

**Additional file 9: Fig. S9.** Subgroup analysis by anesthetic drugs for the brain water content.

**Additional file 10: Fig. S10.** Subgroup analysis by the method of SAH induction for the brain water content.

**Additional file 11: Fig. S11.** Subgroup analysis by the source of MSCs for the brain water content.

**Additional file 12: Fig. S12.** Subgroup analysis by the type of MSCs for the brain water content.

**Additional file 13: Fig. S13.** Subgroup analysis by the type of MSCs-derived therapies for the brain water content..

## Data Availability

The original contributions presented in the study are included in the article and supplementary materials. Further inquiries can be directed to the corresponding author.

## Abbreviations

SAH: Subarachnoid hemorrhage
EBI: Early brain injury
BBB: Blood–brain barrier
CVS: Cerebral vasospasm
DCI: Delayed cerebral ischemia
MSCs: Mesenchymal stem cells
EVs: Extracellular vesicles
SD: Standard deviation
SE: Standard error
SMD: Standardized mean difference
CI: Confidence interval
PRISMA: Preferred reporting items for systematic review and meta-analyses
IV: Intravenous
ICV: Intracerebroventricular
UC-MSCs: Umbilical cord mesenchymal stem cells
BM-MSCs: Bone marrow mesenchymal stem cells
IN: Intranasal
BDNF: Brain derived neurotrophic factor
