# Supplementary figures and images for: Mesenchymal stem cells-derived therapies for subarachnoid hemorrhage in preclinical rodent models: a meta-analysis
Source: Stem Cell Res Ther. 2022 Jan 29;13:42. doi: 10.1186/s13287-022-02725-2 (PMC8800223; doi:10.1186/s13287-022-02725-2)

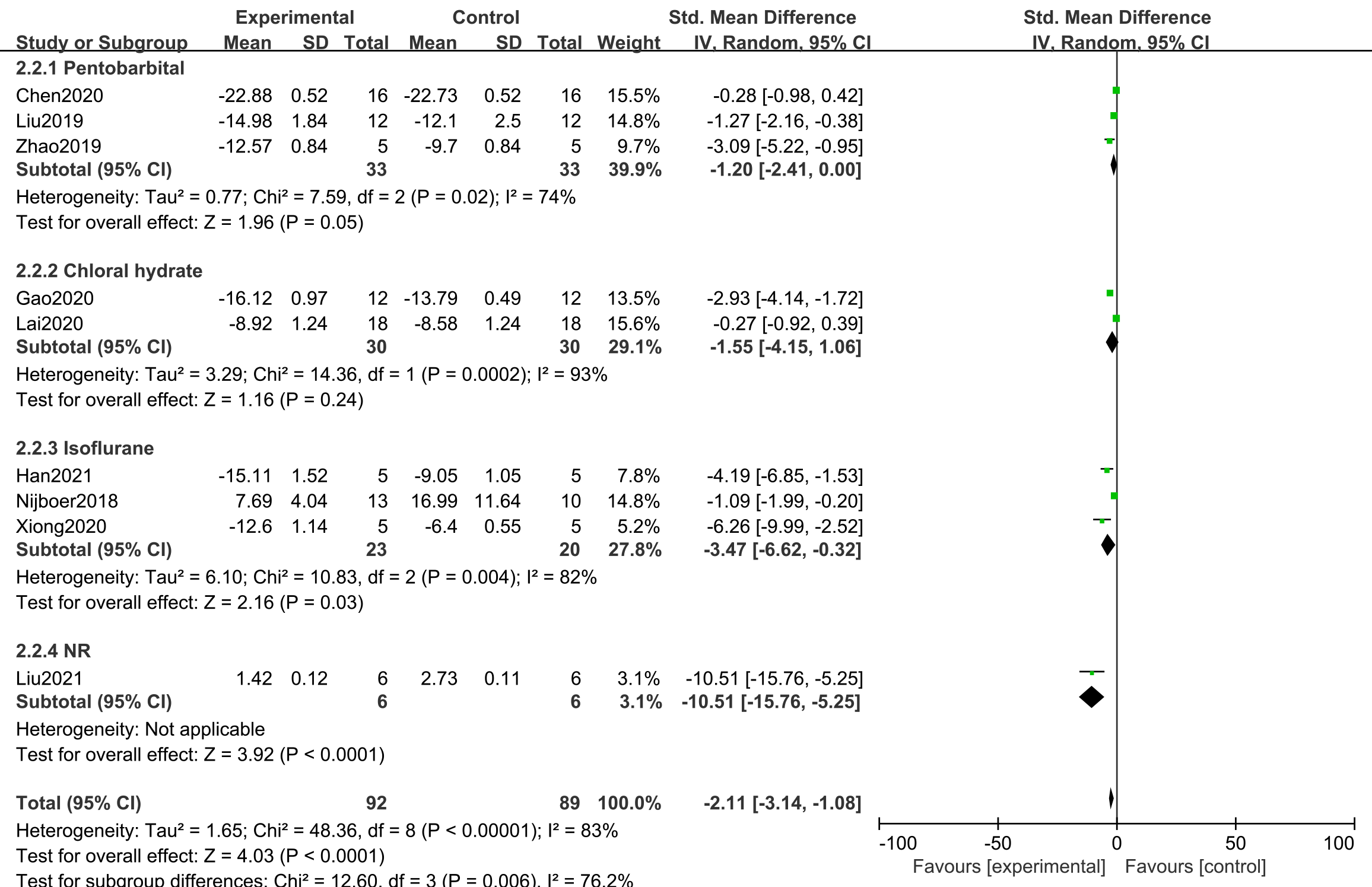

Supplement: Supplementary file 1 — Additional file 1: Fig. S1. Subgroup analysis by anesthetic drugs for the neurobehavioral score. [file 13287_2022_2725_MOESM1_ESM.tif]

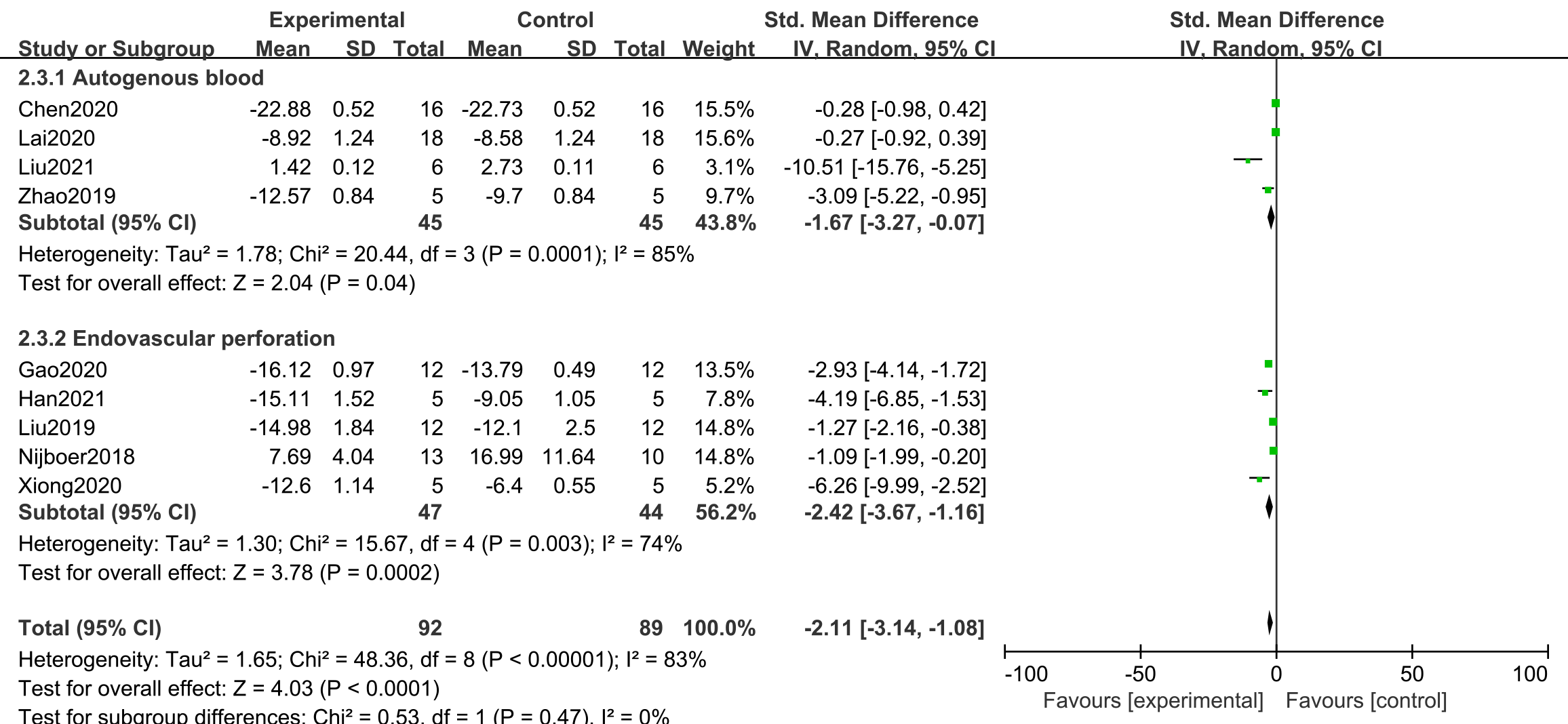

Supplement: Supplementary file 2 — Additional file 2: Fig. S2. Subgroup analysis by the method of SAH induction for the neurobehavioral score. [file 13287_2022_2725_MOESM2_ESM.tif]

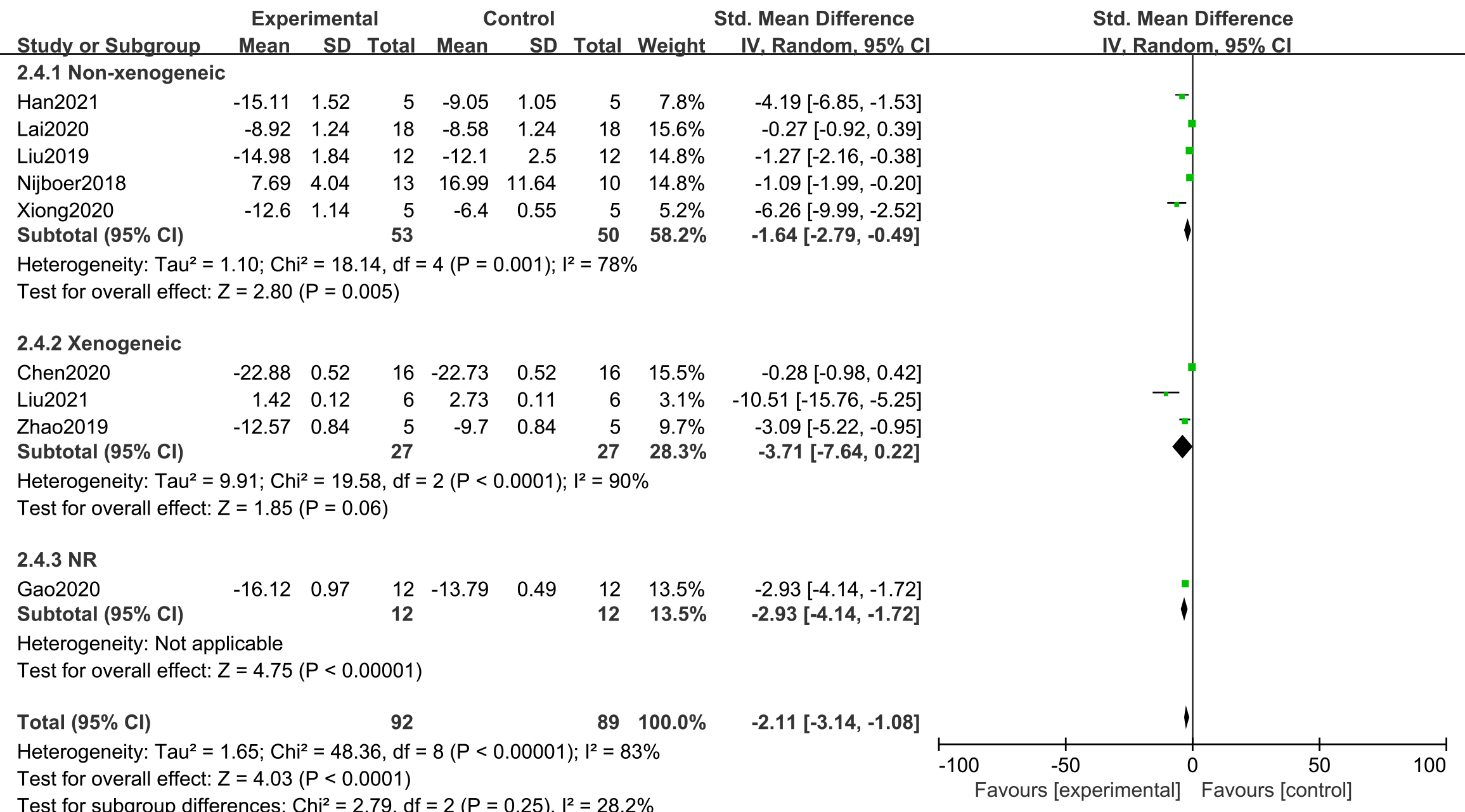

Supplement: Supplementary file 3 — Additional file 3: Fig. S3. Subgroup analysis by the source of MSCs for the neurobehavioral score. [file 13287_2022_2725_MOESM3_ESM.tif]

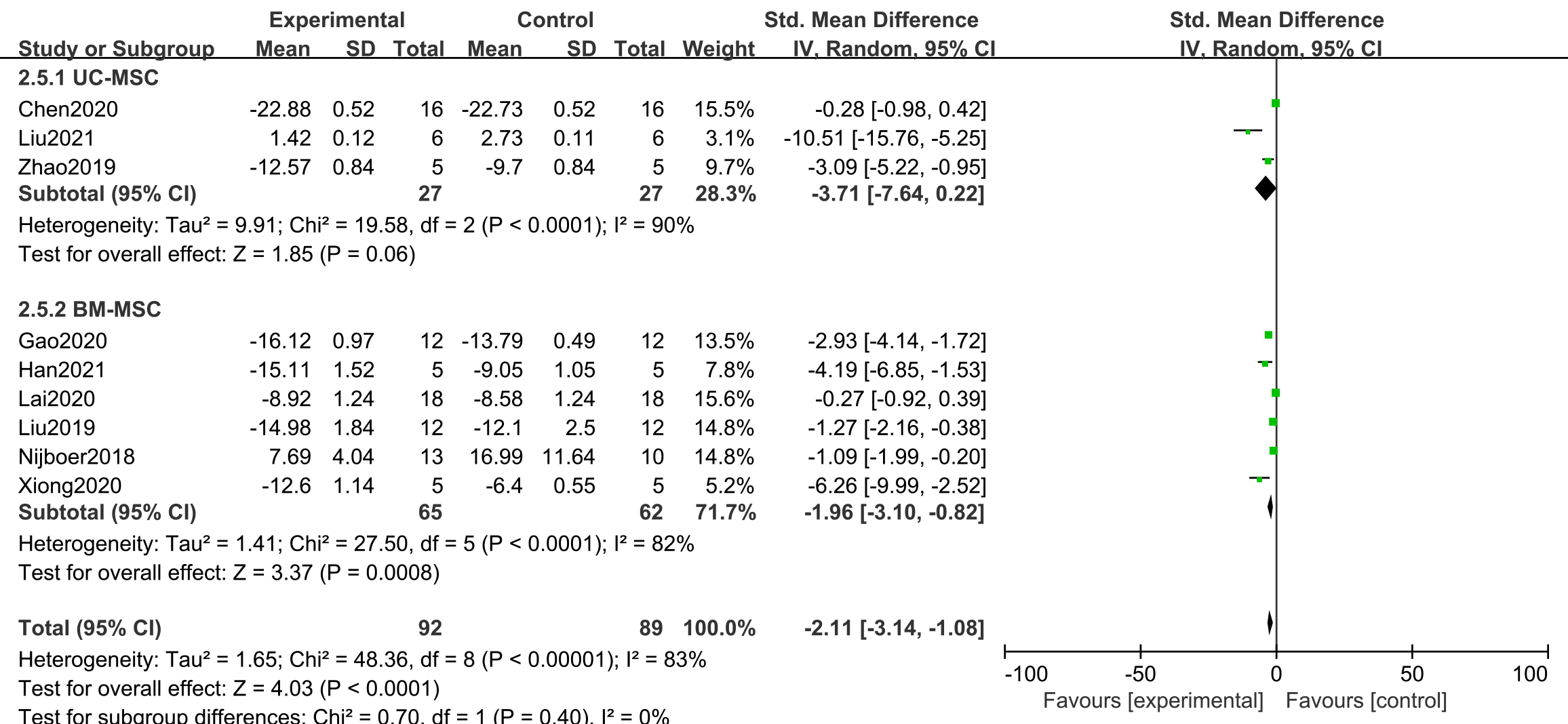

Supplement: Supplementary file 4 — Additional file 4: Fig. S4. Subgroup analysis by the type of MSCs for the neurobehavioral score. [file 13287_2022_2725_MOESM4_ESM.tif]

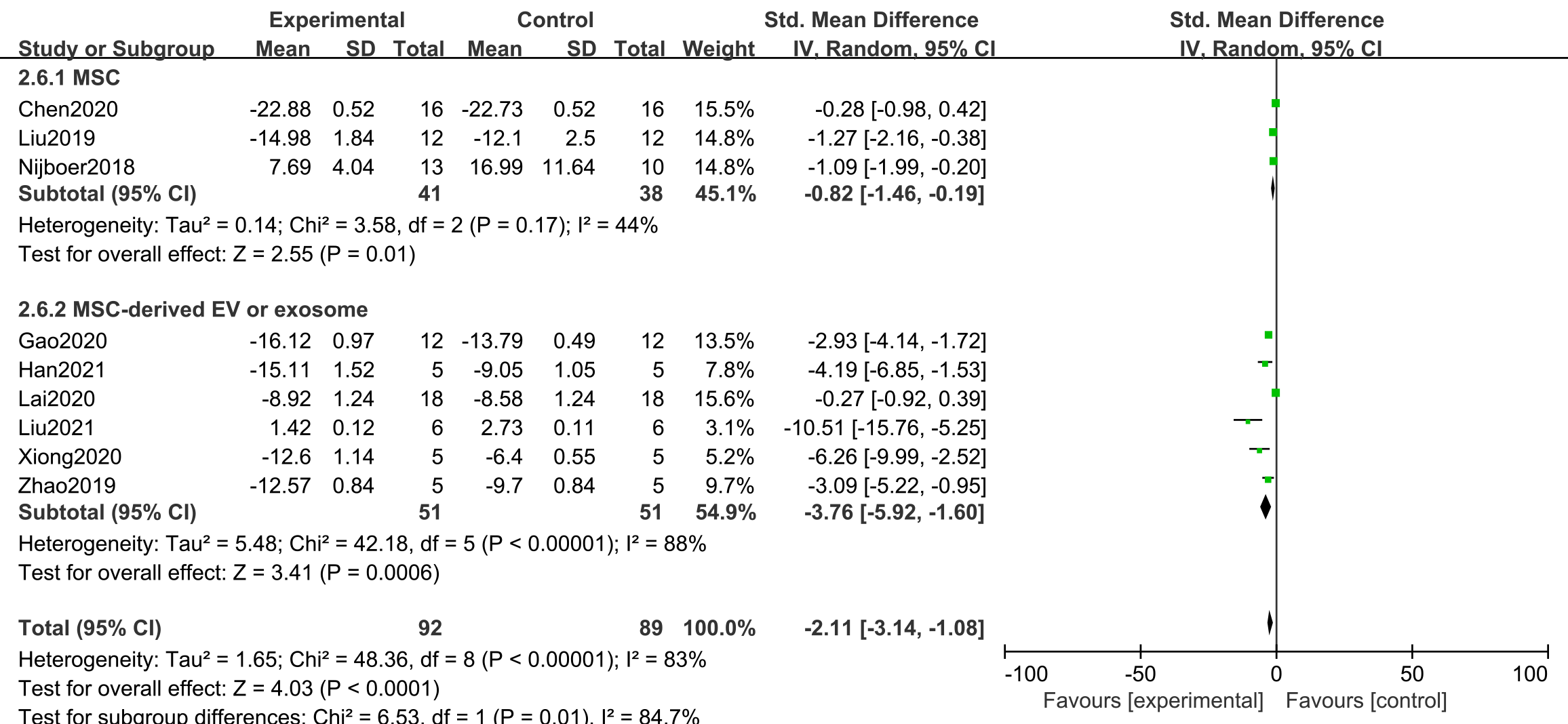

Supplement: Supplementary file 5 — Additional file 5: Fig. S5. Subgroup analysis by the type of MSCs-derived therapies for the neurobehavioral score. [file 13287_2022_2725_MOESM5_ESM.tif]

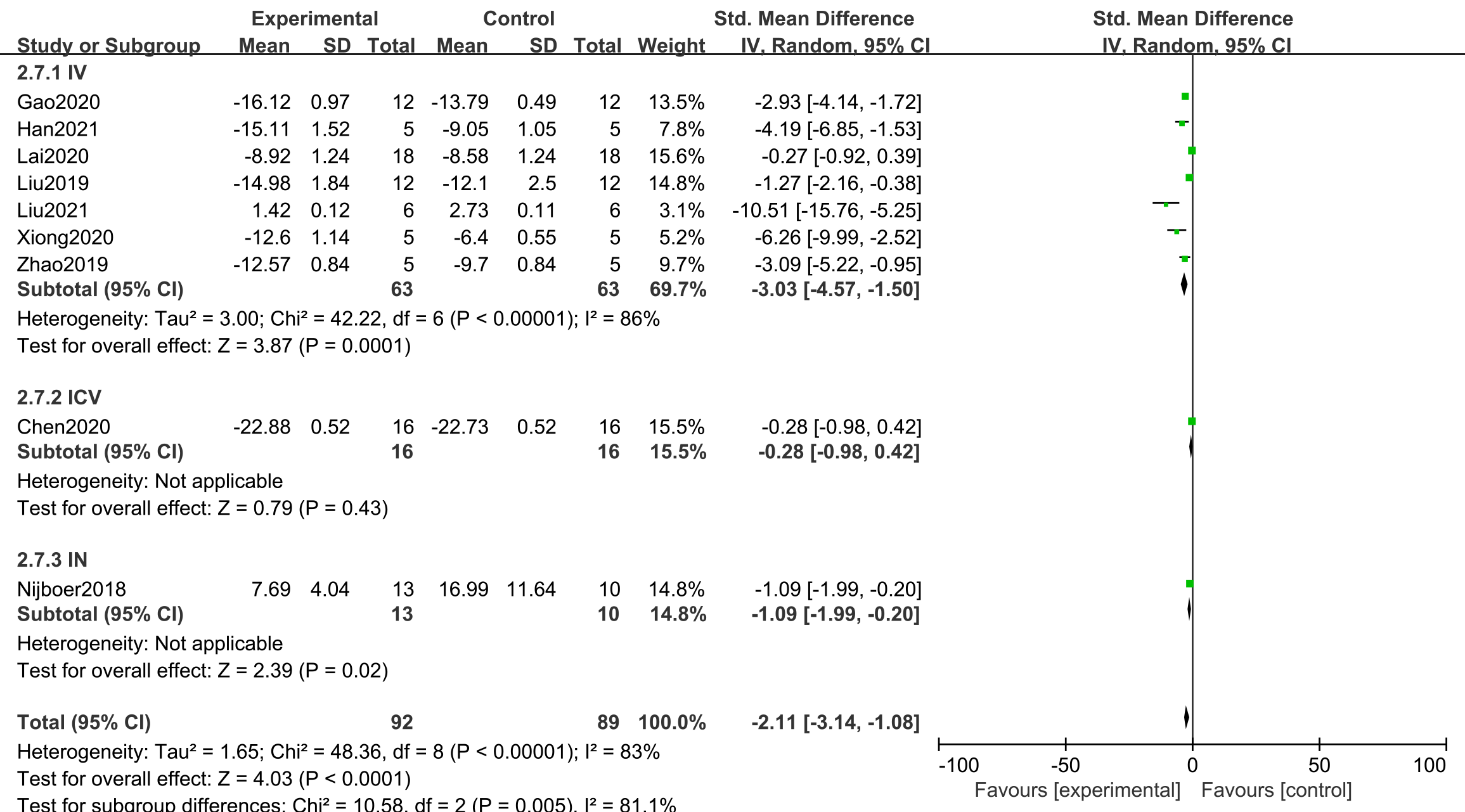

Supplement: Supplementary file 6 — Additional file 6: Fig. S6. Subgroup analysis by delivery route for neurobehavioral score. [file 13287_2022_2725_MOESM6_ESM.tif]

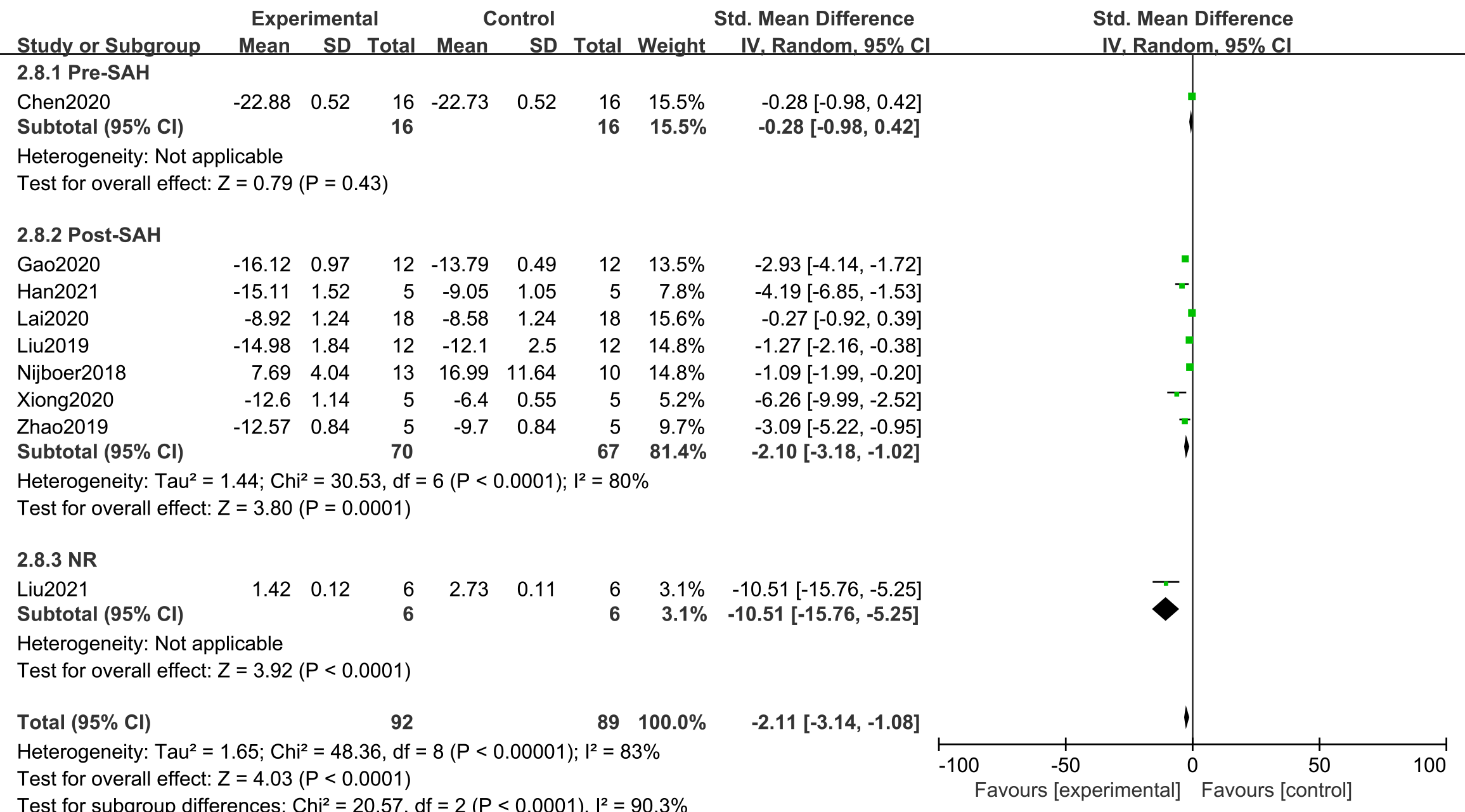

Supplement: Supplementary file 7 — Additional file 7: Fig. S7. Subgroup analysis by administration time for the neurobehavioral score. [file 13287_2022_2725_MOESM7_ESM.tif]

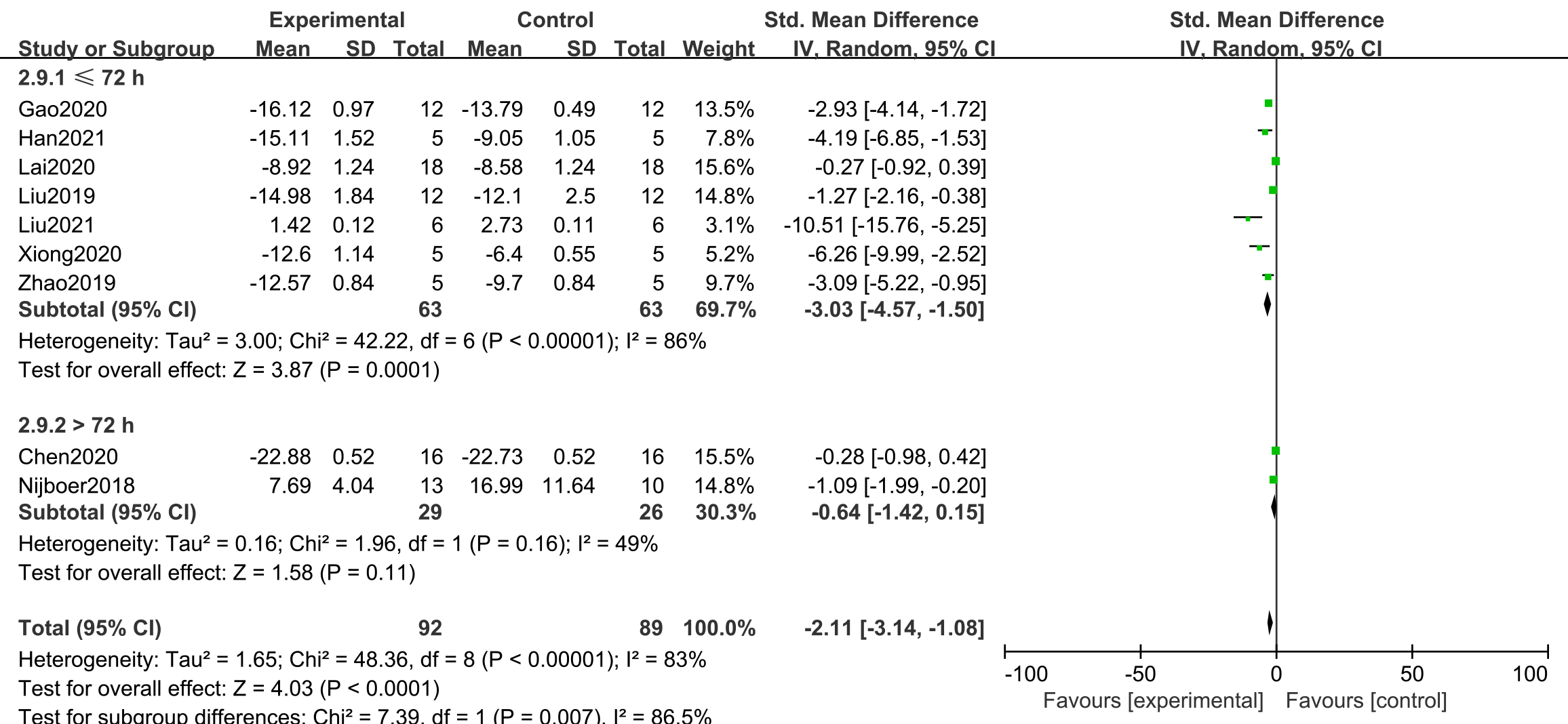

Supplement: Supplementary file 8 — Additional file 8: Fig. S8. Subgroup analysis by assessment time for the neurobehavioral score. [file 13287_2022_2725_MOESM8_ESM.tif]

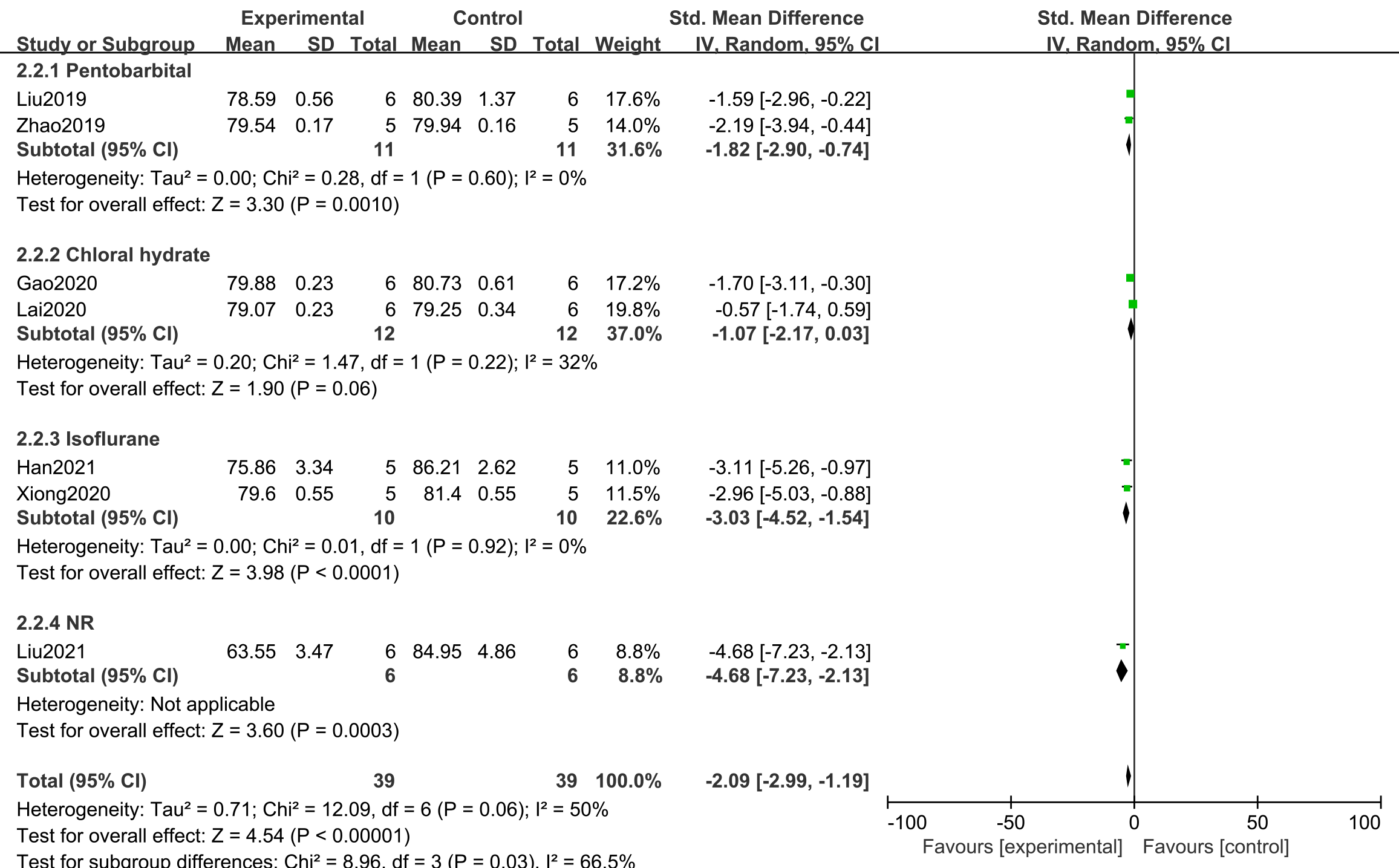

Supplement: Supplementary file 9 — Additional file 9: Fig. S9. Subgroup analysis by anesthetic drugs for the brain water content. [file 13287_2022_2725_MOESM9_ESM.tif]

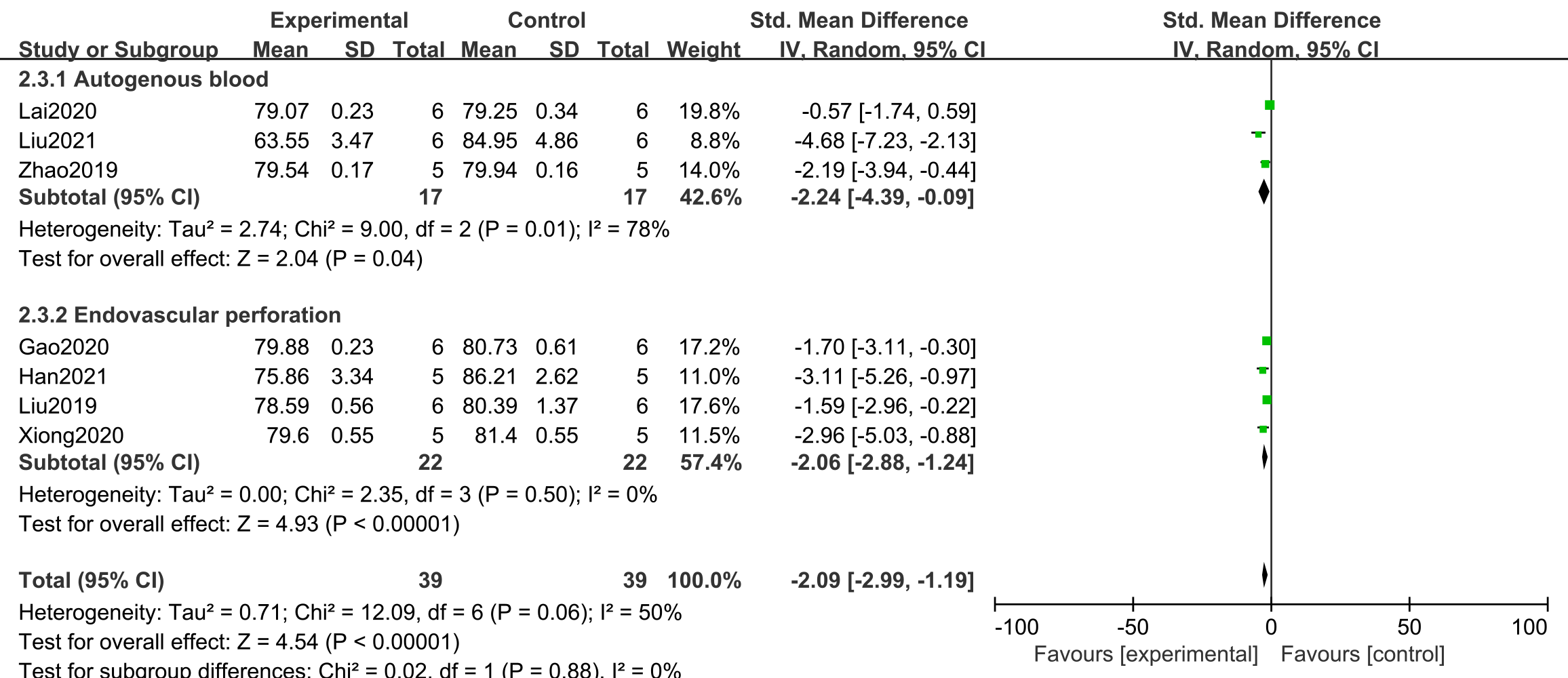

Supplement: Supplementary file 10 — Additional file 10: Fig. S10. Subgroup analysis by the method of SAH induction for the brain water content. [file 13287_2022_2725_MOESM10_ESM.tif]

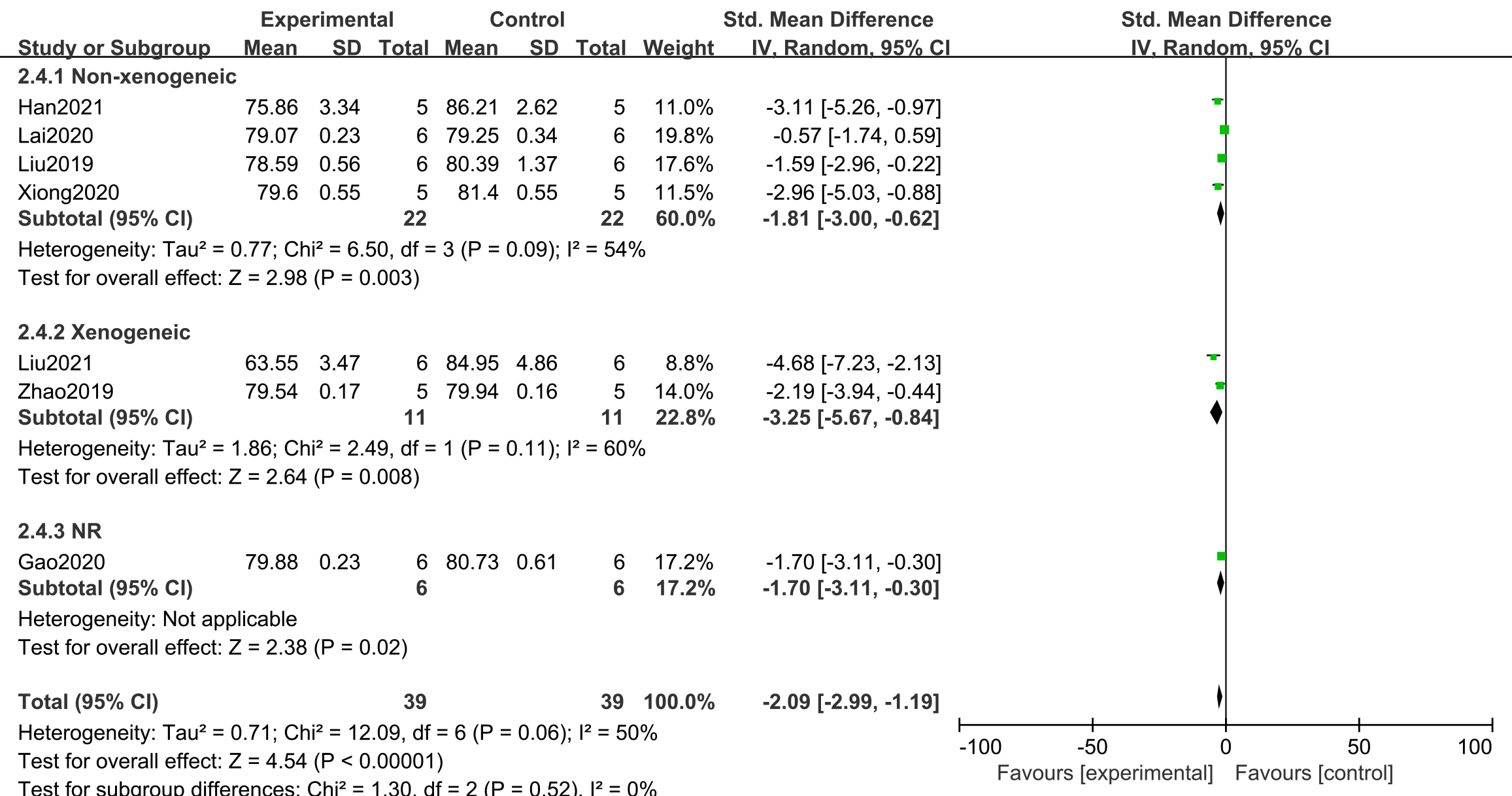

Supplement: Supplementary file 11 — Additional file 11: Fig. S11. Subgroup analysis by the source of MSCs for the brain water content. [file 13287_2022_2725_MOESM11_ESM.tif]

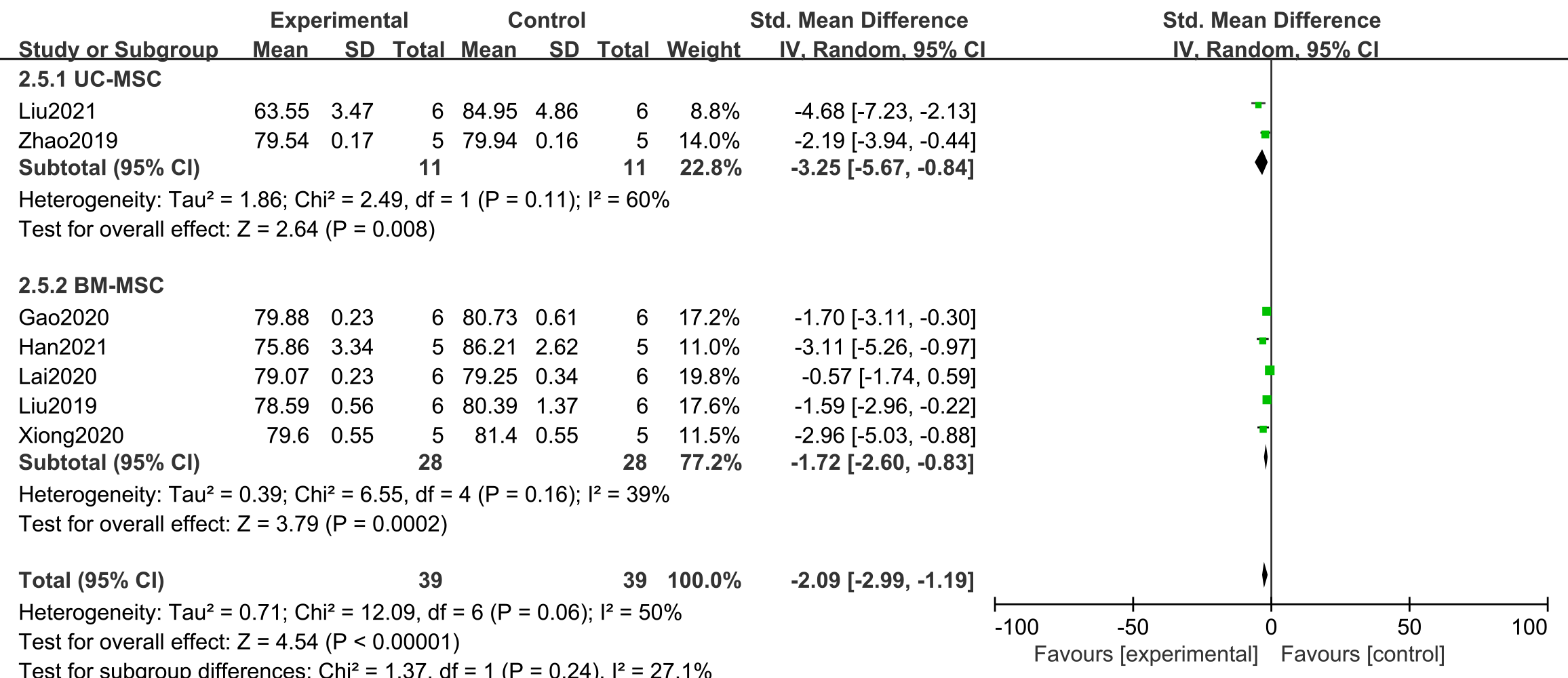

Supplement: Supplementary file 12 — Additional file 12: Fig. S12. Subgroup analysis by the type of MSCs for the brain water content. [file 13287_2022_2725_MOESM12_ESM.tif]

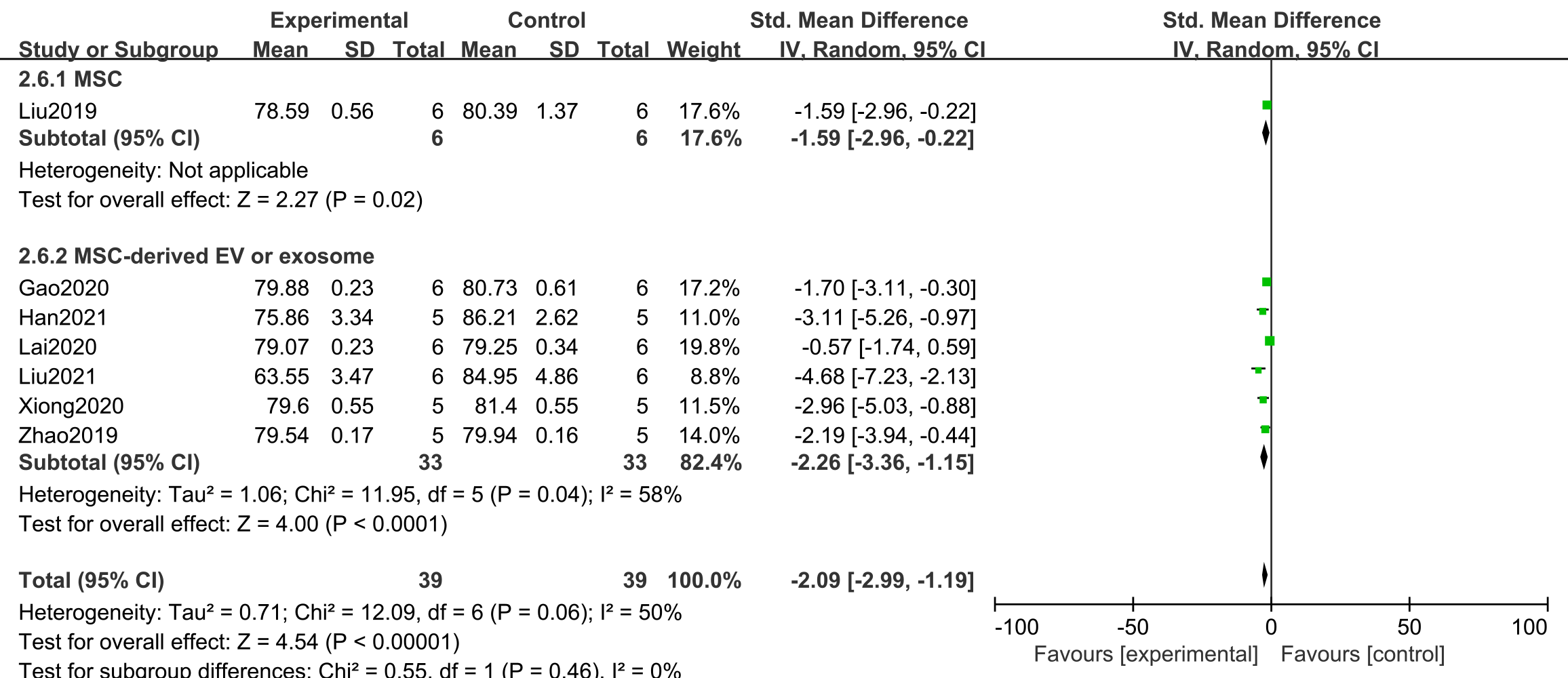

Supplement: Supplementary file 13 — Additional file 13: Fig. S13. Subgroup analysis by the type of MSCs-derived therapies for the brain water content.. [file 13287_2022_2725_MOESM13_ESM.tif]
